# Health‐related quality of life in Norwegian adults with Fabry disease: Disease severity, pain, fatigue and psychological distress

**DOI:** 10.1002/jmd2.12240

**Published:** 2021-07-16

**Authors:** Hege Kampen Pihlstrøm, Mina Susanne Weedon‐Fekjær, Birgitte Leisner Bjerkely, Charlotte von der Lippe, Kristin Ørstavik, Per Mathisen, Ketil Heimdal, Trond Geir Jenssen, Dag Olav Dahle, Olga Karin Solberg, Solrun Sigurdardottir

**Affiliations:** ^1^ Department of Surgery, Inflammation Medicine and Transplantation, Section of Nephrology Oslo University Hospital, Rikshospitalet HF Oslo Norway; ^2^ Centre for Rare Disorders, Oslo University Hospital, Rikshospitalet HF Oslo Norway; ^3^ Department of Neurology, Section for Rare Neuromuscular disorders Oslo University Hospital, Rikshospitalet HF Oslo Norway; ^4^ Department of Cardiology Oslo University Hospital, Rikshospitalet HF Oslo Norway; ^5^ Department of Medical Genetics Oslo University Hospital, Rikshospitalet HF Oslo Norway; ^6^ Institute of Clinical Medicine, Faculty of Medicine, University of Oslo Oslo Norway

**Keywords:** depression, DS3, Fabry, fatigue, HRQOL, pain

## Abstract

Health‐related quality of life (HRQOL) is reduced in Fabry disease (FD) and associated with clinical disease manifestations, but few have used Fabry‐specific severity scores to study how disease burden interferes with quality of life. We investigated how the Fabry DS3, consisting of four somatic domains and one patient‐reported item, associates with HRQOL, while also evaluating fatigue, pain and psychological distress as possible predictors. Thirty‐six adults with FD completed the Short‐form Health Survey (SF‐36), the hospital anxiety and depression scale (HADS), the brief pain inventory (BPI) and reported fatigue on a visual analog scale. Clinical data were collected from the last multidisciplinary hospital visit. Using correlation and hierarchical linear regression analyses, we examined associations between demographic, clinical and self‐reported predictors and the SF‐36 physical (PCS) and mental (MCS) component summary scores. Males scored lower than the general population in all SF‐36 domains (*P* < .05). General health and social functioning were reduced in females. Before including self‐reported symptom scores, DS3 showed associations with PCS (*P* = .009). Our fully adjusted model explained 66% of the variation in PCS, where education (*P* = .040) and fatigue (*P* = .002) retained significance. With HADS depression score (*P* = .001) as the sole significant factor, our regression model explained 56% of the variation in MCS. The DS3 score has implications for HRQOL in FD. Low education and fatigue represent major barriers to physical well‐being, while depression strongly influences mental quality of life. Fatigue should be recognized as an important endpoint in future FD trials. Increased efforts to diagnose and treat affective disorders are warranted.

**Synopsis**

Disease severity as measured by DS3, educational level and fatigue are major explanatory factors for physical health‐related quality of life in Fabry disease, while depression, an underdiagnosed and undertreated problem, has a strong effect on mental quality of life.

## 1 INTRODUCTION

Lysosomal storage disorders are rare genetic diseases affecting the metabolism and clearance of sphingolipids. Of these, the X‐linked Fabry disease (FD; OMIM 301500) is the most common (1:40 000 males).^1^ Mutations in the *GLA* gene cause deficiency of α‐galactosidase (α‐GAL), and globotriaosylceramide (Gb3) accumulates in lysosomes causing progressive tissue damage due to inflammation, ischemia, hypertrophy and fibrosis.^2^ Early FD manifestations include neuropathic pain,^3^ angiokeratomas^4^ and gastrointestinal symptoms.^5^ Left untreated, classical FD in males results in progressive renal, cardiac and cerebrovascular disease and premature death.^6^ Females have residual enzyme activity but may still develop clinical disease,^7^ though in average with a milder phenotype.^8^, ^9^ Some *GLA*‐variants cause nonclassical late‐onset FD with a milder course, also in males.^10^ Plasma globotriaosylsphingosine (lyso‐Gb3) may serve as biomarker of disease activity and progression.^11^, ^12^ Since 2003, the standard treatment for FD has been intravenous enzyme replacement therapy (ERT),^13^, ^14^ but in 2016 oral treatment with the pharmacological chaperone, migalastat, became an option for patients with amenable mutations.^15^

Patients with FD experience physical disabilities and have a shortened life expectancy. Health‐related quality of life (HRQOL) is also reduced compared with the general population,^16^, ^17^ especially from the third decade onward.^18^ A major challenge when studying HRQOL in FD is to stratify for the relative impact of the different medical conditions that may influence life satisfaction. HRQOL is lower in patients suffering from Fabry complications,^19^ such as stroke, cardiovascular disease^16^, ^20^ and renal dysfunction.^21^ Patient‐reported symptoms may also predict HRQOL.^22^ Though inconsistent, published data suggest an effect of ERT on HRQOL.^16^, ^23^, ^24^, ^25^

Attempts to develop disease‐specific instruments to measure the total morbidity caused by FD have resulted in two validated scoring systems, the Mainz Severity Score Index (MSSI)^26^ and the Fabry DS3.^27^ The DS3 would be the easiest to implement in routine clinical practice: composed of four clinical domains (peripheral nervous system, renal, and cardiac, each with three items; central nervous system with two items) and a patient‐reported domain with one item. Few have applied disease severity scores in HRQOL studies in FD.^19^ The current study aimed to explore potential associations between FD severity as measured by the DS3 and different aspects of HRQOL.

Patients, however, often complain about fatigue, pain and psychological distress.^28^, ^29^, ^30^ Starting in childhood,^31^ acroparesthesias affect 60% to 80% of adults.^32^ Exacerbating factors include fever, exercise, fatigue or stress.^3^, ^33^ Anxiety and depression is also common (42%‐46%),^34^ especially in males.^35^, ^36^, ^37^ In light of this, we also sought to evaluate the influence of *pain* on HRQOL, as measured by the brief pain inventory (BPI),^38^ *fatigue* reported on a visual analog scale (VAS‐F) and *psychological symptoms* as measured by the Hospital and Anxiety Scale (HADS).^39^

## 2 METHODS

### 2.1 Study population

By November 2019, 110 individuals were known to have FD in Norway. Fifty‐six patients were attending regular follow‐up visits at Oslo University Hospital Rikshospitalet. Irrespective of ERT treatment status, most patients are scheduled for a yearly 3 to 5 days of multidisciplinary work‐up, which involves a broad range of medical subspecialties. Visits include symptom assessment (pain, sweating, fatigue, digestive or respiratory problems), clinical examination (height, weight, vital signs, organ status), as well as supplementary investigations evaluating both early and more established clinical disease manifestations, including measured renal function, resting and 24‐hours electrocardiogram, echocardiography, magnetic resonance imaging brain and heart scans, pulmonary function test and precerebral artery ultrasound. Patients are seen by neurologist, cardiologist and nephrologist on a regular basis, while consultations with other specialists are scheduled on demand.

For inclusion in the current study participants should be >18 years, diagnosed with a pathogenic mutation in the *GLA* gene, have undergone at least one multidisciplinary Fabry‐assessment in the period 2006 to 2020 and have signed informed consent. Six individuals whose *GLA* variant was not classified as pathogenic were excluded. Seventy‐eight percent of 46 eligible patients signed written informed consent. There were no differences in sex distribution (*P* = .412) or age (*P* = .827) between participants and nonresponders. All participants were of white European descent.

Study information and questionnaires were distributed by mail, and self‐reported data were collected between March and August 2020.

### 2.2 Ethical statement

All protocols and methods were approved by the Norwegian Regional Committee for Medical Research Ethics in South‐Eastern Norway, REK‐Sør Øst (permit no. 31513) and the Institutional Data Protection Authority (PVO).

### 2.3 Study assessments

#### 2.3.1 Laboratory and clinical data

For participants diagnosed in the period 2006 to 2017, α‐GAL activity in plasma (ref. ≥2.3 nkat/L) was analyzed at Sahlgrenska University Hospital (Gothenburg, Sweden). Since 2017, we have used Centogene GmbH (Rostock, Germany), who quantifies enzyme activity (ref. ≥15.3 μmol/L/h) in dried whole blood spots. The remainder of biochemical analyses, vital signs and organ function measurements were collected from hospital files from the most recent Fabry work‐up (May 2018‐September 2020). Lyso‐Gb3 was measured as concentration (ref. ≤1.8 ng/mL) in dried blood spots by Centogene GmbH. Measured GFR (mL/min/1.73 m^2^) was obtained by calculating the rate of reduction in plasma activity of technetium in four samples taken over a 3 to 4 hour period after intravenous injection, and results were standardized to body surface area. Small fiber neuropathy was diagnosed based on registration of pathological thermal thresholds in extremities. Hearing was tested with audiometry at least once in the period 2006 to 2020. Functional limitation due to dyspnea or angina was assessed using New York Heart Association (NYHA) Functional Classification: class 1 = no symptoms, class 2 = mild symptoms, class 3 = marked limitations and class 4 = severe limitations.^40^

#### 2.3.2 Disease severity measure

Clinical data necessary for calculating the DS3 were found in the medical files from the most recent Fabry follow‐up visit. A study nurse collected information necessary for the completion of the peripheral nervous system domain and the patient reported domain via telephone consultation. Each domain score is obtained by averaging scores for all domain items, rendering a maximum global DS3 score of 32 (sum of all averaged domains).^27^ Higher DS3 scores indicate more severe disease.

#### 2.3.3 Self‐reported HRQOL

The main outcome measure used was *The Short Form (SF‐36) Health Survey*, a self‐reported instrument evaluating the impact of disease on activities of daily living and quality of life. A license from Quality Metric was used for scoring (License Agreement QM049911). The SF‐36 contains 36 items and measures 8 health domains of physical and emotional health. For each domain, the possible score is 0 to 100, where higher scores indicate better health. Age‐ and sex‐matched control groups are available for comparison.^41^ SF‐36 scores may be summarized by aggregating the physical and mental domain subscales into two constructs: physical component summary (PCS) and mental component summary (MCS) scores. The relative weight of each subscale is determined by factor analysis.^42^ The PCS, reflecting physical morbidity and adaptation to disease and the MCS, reflecting psychological morbidity and adaptation, are normalized to a general population mean of 50 and a standard deviation of 10.

Average SF‐36 domain scores for 2118 individuals from the general population in Norway, collected in 2015, were used as country‐specific reference.^43^

#### 2.3.4 Self‐reported physical function and psychological symptoms

Pain was assessed using the validated Norwegian translation of the BPI,^38^ a questionnaire originally developed for the evaluation of cancer pain,^44^ but validated also in noncancer populations.^45^ *Pain severity* was assessed using scores for average pain in the last 24 hours. *Pain interference* was scored as the mean of the seven interference items: general activity, walking, work, sleep, mood, relations with others and enjoyment of life.

As a supplement to BPI, pain severity during the previous 7 days was measured using a visual analog scale for pain (VAS‐P). VAS‐P was rated on a 100‐mm horizontal line, ranging from 0 (no pain) to 100 (very severe pain).

Severity of fatigue during the previous 7 days was measured using a visual analog scale for fatigue (VAS‐F) and is rated on a 100‐mm horizontal line, ranging from 0 (no fatigue) to 100 (very severe fatigue).

Since no disease‐specific instrument exists for evaluating psychological symptoms in FD,^46^ we used the hospital anxiety and depression scale (HADS),^39^ a generic instrument widely used to measure depression and anxiety in various somatic and psychiatric populations.^47^ HADS has two subscales (anxiety, depression), each consisting of seven items rated on a 4‐point scale from 0 (no symptom) to 3 (severe symptoms). The cutoff score >7 is used as threshold for each subscale, indicating a mild, but clinically significant, level of depression (HADS‐D) or anxiety (HADS‐A).

### 2.4 Statistical analyses

Statistical analyses were performed using IBM SPSS 25 Statistics. We present mean and SD or median and interquartile range for continuous data and percentages for categorical data. Sex differences in clinical parameters and baseline characteristics were investigated using *t* test for normally distributed variables and Mann‐Whitney *U* test for non‐normal variables. For categorical parameters, chi‐square/Fisher's exact test was used as appropriate. Both SF‐36 component scores (PCS and MCS) appeared normally distributed (by Kolmogorov‐Smirnov test and histograms).

Bivariate two‐tailed Pearson (for interval scales), Spearman (for ordinal scales) and Eta (for string variables with >2 levels) coefficients were calculated for associations among the SF‐36 components scores, demographics, medical and self‐reported variables. Correlations with coefficients 0‐0.19 was regarded as very weak, 0.2‐0.39 as weak, 0.40‐0.59 as moderate, 0.6‐0.79 as strong and 0.8‐1 as very strong.^48^ Due to multiple comparisons and small sample size, *P* < .001 was applied to counteract type I errors.

We performed hierarchical linear regression with PCS and MCS as dependent variables using age, sex, education, disease severity (global DS3), fatigue (VAS‐F) and depression (HADS‐D) as explanatory variables. These covariates were selected because they have been identified as influential for HRQOL in previous research. BPI interference score was strongly correlated with VAS‐F (*r* = 0.639), introducing multicollinearity. Since global DS3 already incorporates a scale for pain intensity, we refrained from including a second pain score in the model. Bootstrapping was used to optimize model stability due to small sample size and non‐normal distribution of some explanatory variables. We report adjusted *R* square values.

A two‐sided *P*‐value of <.05 was used as indicator for statistical significance. Our regression model rendered tolerance levels >0.5 for all variables and no variable inflated factor >2. Residuals showed a normal distribution. For outlier diagnostics, we calculated Mahalanobis distances, finding no value exceeding the limit (22.46 with 6 degrees of freedom and critical alpha value .001).

## 3 RESULTS

### 3.1 Patient characteristics

A list of the *GLA* mutations represented in our study cohort is available in Suppl. Table 1. Fifty‐six percent of males (n = 9) were categorized as having a classical phenotype, defined as α‐GAL activity <2% of reference and early debut of clinical symptoms (eg, neuropathic pain or anhidrosis). Table 1 presents the demographic and clinical characteristics of participants at time of inclusion. All males were treated with either ERT or chaperone, while 50% of the females received Fabry‐specific drugs—generally with shorter treatment duration. Out of six patients (only one female) using migalastat, two persons started with chaperone therapy as first Fabry‐specific treatment and three were switched from ERT (2018‐2019), mainly for practical reasons. Verified small fiber neuropathy was more frequent in males (*P* = .002). There were no significant sex differences in terms of disease severity (DS3 domain scores) or pain (BPI, VAS‐P), fatigue (VAS‐F) and psychological symptoms (HADS). Not surprisingly, pain as reported by BPI was mainly present in extremities (Suppl. Table 2). Of note, 22% of the patients scored >7 points on HADS‐D and 36% scored >7 points on HADS‐A.

**TABLE 1:** Demographics, clinical characteristics and symptom scores of the sample (N = 36)

	Total n = 36	Men n = 16 (44%)	Women n = 20 (56%)	*P*‐value**
Demographics				
Age at inclusion (years, range 21‐78)	49.1 (15.1)	50.2 (13.1)	48.2 (16.8)	.701
Age at diagnosis / first clinical visit	44.3 (13.9)	43.3 (9.4)	45.0 (16.8)	.707
Education >12 years	22 (61%)	10 (63%)	12 (60%)	.878
Marital status single	11 (31%)	4 (25%)	7 (35%)	.718
Currently employed/student	20 (56%)	10 (63%)	10 (50%)	.453
Clinical variables				
α‐GAL activity <2% of ref.	9 (25%)	9 (56%)	0 (0%)	**<.001**
Cornea verticillata (n = 34)	19 (56%)	5 (36%)	14 (70%)	**.048**
Cardiomyopathy (IVS ≥1.15 cm/ LV dilat.)	17 (47%)	8 (50%)	9 (45%)	.765
EF <50%	6 (17%)	5 (31%)	1 (5%)	.069
NYHA class ≥2	27 (75%)	14 (88%)	13 (65%)	.245
Pacemaker/ICD implant	5 (14%)	3 (19%)	2 (10%)	.637
History of cerebral ischemic event	11 (31%)	6 (38%)	5 (25%)	.483
Small fiber neuropathy (n = 34)	14 (41%)	11 (69%)	3 (17%)	**.002**
Measured GFR (mL/min/1.73 m^2^)	72.9 (26.4)	68.6 (30.2)	76.4 (23.3)	.388
RRT (kidney transplant/dialysis)	4 (11%)	4 (27%)	0 (0%)	**.026**
Reduced hearing/tinnitus	15 (43%)	8 (50%)	7 (37%)	.433
Lyso‐Gb3 (ng/mL)*	4.9 (7.5)	5.5 (24.7)	4.9 (6.3)	.301
Current ERT/chaperone treatment	26 (72%)	16 (100%)	10 (50%)	**.001**
Treatment duration (years)*	4 (13)	9 (15)	1 (10)	**.031**
Comorbidity				
Gastrointestinal disease^a^	10 (28%)	2 (13%)	8 (40%)	.133
Diabetes mellitus (type 1 or 2)	1 (3%)	0 (0%)	1 (5%)	**1.000**
History of cancer^b^	5 (14%)	2 (13%)	3 (15%)	1.000
Chronic obstructive pulmonary disease^c^	9 (25%)	6 (38%)	3 (15%)	.146
Coronary artery stenosis^d^	3 (8%)	2 (13%)	1 (5%)	**.582**
DS3 disease domains				
Total DS3 (max score 80)*	18 (19)	25.5 (31)	16 (15)	**.077**
Averaged DS3 (max score 32)*	7.5 (10.5)	9.6 (13.3)	7.0 (8.0)	.095
PNS (max score 12)*	6.5 (4)	6 (7)	7.5 (4)	.694
Renal (max score 24)*	4 (12)	8 (16)	0.5 (4)	.053
Cardiac (max score 24)*	4.5 (10)	7.5 (16)	2 (9)	**.083**
CNS (max score 16)*	1 (7)	1 (8)	0 (5)	.459
Patient‐reported (max score 4)*	2 (2)	2 (2)	1 (3)	.189
Pain/fatigue (max scores 10)				
BPI pain interference	2.6 (2.4)	2.9 (3.0)	2.4 (2.0)	.620
BPI pain severity average	3.8 (2.6)	3.2 (2.6)	4.2 (2.5)	.260
VAS‐P (last 7 days)*	2.0 (5.8)	1.6 (7.0)	2.0 (5.5)	.694
VAS‐F (last 7 days)*	6.9 (4.7)	7.3 (9.8)	6.8 (5.4)	.962
Anxiety and depression				
HADS total score (max 42)	10.0 (6.0)	10.6 (7.6)	9.6 (4.5)	.654
HADS anxiety (max 21)	5.7 (3.1)	5.5 (3.7)	6.0 (2.5)	.672
HADS depression (max 21)*	2.5 (6)	3 (8)	2 (6)	.502
HADS depression score >7	8 (22%)	5 (31%)	3 (15%)	.422
HADS anxiety score >7	13 (36%)	6 (38%)	7 (35%)	.968

*Notes*: Values are mean (SD), median (IR)* or n (%).

Abbreviations: BPI, brief pain inventory; CNS, central nervous system; EF, ejection fraction; ERT, enzyme replacement therapy; GFR, glomerular filtration rate; HADS, Hospital Anxiety and Depression Score; ICD, intracardiac defibrillator; IVS, intraventricular septum; LV, left ventricle; Lyso‐Gb3, globotriaosylsphingosine; NYHA, New York Heart Association; PNS, peripheral nervous system; RRT, renal replacement therapy; VAS‐F; fatigue on visual analog scale; VAS‐P, pain on visual analog scale; α‐GAL, alpha galactosidase.

^a^ Chronic inflammatory/autoimmune conditions affecting the bowels, liver, gall bladder or exocrine pancreas or acute conditions in demand of hospitalization, for example, ulcerations in the GI‐tract, bowel obstructions, diverticulitis (not including surgery for appendicitis).

^b^ Not including basalioma/spinocellular skin cancer.

^c^ History of asthma in childhood/adolescence, obstructive respiratory pattern (FEV1/FVC <70%) documented by spirometry at Fabry follow‐up or regular use of bronchodilators on this indication.

^d^ A documented narrowing of ≥50% of lumen by coronary angiography, and/or a history of percutaneous transluminal angioplasty.

** Mann‐Whitney *U*‐test, *t* test, chi‐square test, Fisher's exact test as found appropriate.

Bold values indicate significant associations by the <0.05 threshold.

### 3.2 Comparison of HRQOL in persons with FD vs the general Norwegian population

Scores for each SF‐36 domain in males and females with FD are presented in Table 2, together with values from a general population sample.^43^ *T* tests for comparisons with the Norwegian normal population were performed for domain scores. Males with FD scored significantly lower in all domains (all *P*‐values <.05). Females with FD scored lower on the General health (*P* = .001) and Social functioning (*P* = .004) domains compared with the Norwegian population. Within the FD group, significantly worse scores were found in males than females on the Role emotional domain (*P* = .012).

**TABLE 2:** Health related quality of life in Fabry patients by SF‐36 scores. Sex differences and comparison with the background population

SF‐36 domains	Men			Women			Men vs women in FD
	Gen. pop. 2015 N = 917	FD 2020 N = 16	*P*	Gen. pop. 2015 N = 1091	FD 2020 N = 20	*P*	*P*
Physical functioning	88.1 (17.0)	63.7 (28.7)	**.001**	84.9 (21.0)	73.7 (27.9)	.148	.299
Role physical	78.9 (35.3)	53.5 (34.4)	**.007**	72.6 (39.6)	62.2 (33.5)	.341	.461
Bodily pain	72.1 (25.4)	49.7 (31.0)	**.008**	66.9 (26.5)	56.1 (30.0)	.220	.534
General health	73.4 (20.8)	43.7 (28.7)	**<.001**	72.6 (22.5)	50.8 (26.2)	**<.001**	.447
Vitality	61.9 (18.9)	36.3 (23.9)	**<.001**	57.2 (20.6)	45.0 (24.9)	.059	.297
Social function	89.0 (19.3)	54.7 (37.6)	**<.001**	85.7 (21.6)	65.6 (28.6)	**.004**	.345
Role emotional	89.5 (26.3)	73.4 (18.1)	**.001**	87.4 (28.6)	100 (22.9)*	NA	.012**
Mental health	81.9 (13.8)	70.6 (16.9)	**.016**	79.9 (14.8)	76.5 (12.2)	.438	.234
SF‐36 component summary scales							
Physical component (PCS)	NA	39.8 (13.3)	NA	NA	42.5 (14.6)	NA	.572
Mental component (MCS)	NA	45.5 (9.8)	NA	NA	50.1 (7.7)	NA	.128

*Notes*: Comparisons by *t* test except for: *Median with interquartile range; comparison by *t* test not possible. **Comparison by Mann‐Whitney *U* test.

Abbreviations: MCS, mental component summary; PCS, physical component summary.

### 3.3 Factors associated with HRQOL

As seen in Table 3, educational level, BPI pain interference, BPI average intensity, VAS‐P last 7 days, VAS‐F last 7 days, global DS3 and NYHA class were significantly correlated with PCS (all *P*‐values <.001). HADS scores were the only factors significantly associated with MCS (all *P*‐values <.001).

**TABLE 3:** Bivariate correlations between explanatory variables and SF‐36 summary scores

Explanatory variable	SF‐36 PCS		SF‐36 MCS	
	Corr. coeff	*P*	Corr. coeff	*P*
Age at inclusion (years)	−0.205	.231	0.176	.304
Education >12 years	0.593	**<.001**	−0.246	.148
Currently employed/student	0.390	.019	−0.049	.776
Female sex	0.097	.572	0.258	.128
Marital status single	−0.122	.478	−0.246	.148
α‐GAL activity <2% of lower ref. limit	−0.373	.025	−0.120	.484
Lyso‐Gb3 (ng/mL)*	−0.412	.014	0.118	.500
Current ERT/chaperone treatment	0.199	.245	0.150	.383
Small fiber neuropathy	−0.341	.048	0.060	.736
BPI pain interference*	−0.814	**<.001**	−0.010	.953
BPI pain severity average*	−0.651	**<.001**	0.122	.483
VAS‐P (last 7 days)*	−0.713	**<.001**	0.146	.396
VAS‐F (last 7 days)*	−0.759	**<.001**	−0.153	.373
HADS total score*	−0.262	.128	−0.739	**<.001**
HADS anxiety*	−0.023	.897	−0.616	**<.001**
HADS depression*	−0.456	.006	−0.655	**<.001**
Averaged DS3 score*	−0.619	**<.001**	−0.137	.427
DS3 PNS*	−0.471	.004	−0.102	.554
DS3 renal*	−0.189	.269	0.148	.389
DS3 cardiac*	−0.544	.001	0.150	.383
NYHA class (1‐4)	−0.789	**<.001**	0.134	.873
DS3 CNS*	−0.185	.281	−0.001	.996
DS3 patient reported*	−0.596	**<.001**	−0.270	.111

*Notes*: Pearson/point‐biserial/eta correlation coefficients (*Spearman correlation coefficients for non‐normal distributed or ordinal scale data) between putative explanatory variables and the SF‐36 physical (PCS) and mental (MCS) components scores.

Abbreviations: BPI, brief pain inventory; CNS, central nervous system; ERT, enzyme replacement therapy; HADS, Hospital Anxiety and Depression Score; Lyso‐Gb3, globotriaosylsphingosine; MCS, mental component summary; NYHA, New York Heart Association; PCS, physical component summary; PNS, peripheral nervous system; VAS‐F, fatigue on visual analog scale; VAS‐P, pain on visual analog scale; α‐GAL, alpha galactosidase.

Bold values indicate significant associations by the <0.001 threshold.

### 3.4 Associations between explanatory factors

**FIGURE 1:**
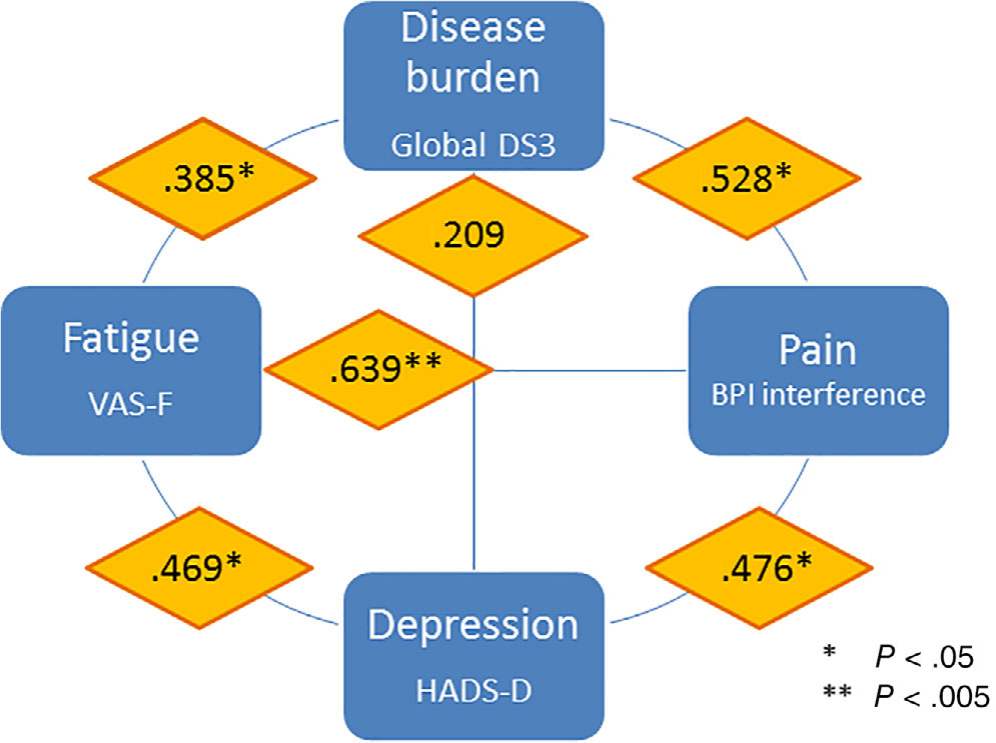
Interrelationship between variables that influence health‐related quality of life (HRQOL) in Fabry disease (FD). Correlations between variables are presented using Spearman coefficients (*ρ*)

The interrelationship between explanatory factors was investigated, and Figure 1 visualizes Spearman correlations between disease burden (DS3), fatigue (VAS‐F), pain (BPI) and depression (HADS‐D). There were moderate‐to‐strong correlations between BPI interference and both global DS3 score (*ρ* = 0.528) and VAS‐F (*ρ* = 0.639). A moderate correlation was found between BPI interference and HADS‐D (*ρ* = 0.476). VAS‐F was weakly correlated with global DS3 (*ρ* = 0.385) and moderately correlated with HADS‐D (*ρ* = 0.469). HADS‐D and global DS3 were not significantly correlated. Not shown in the figure, a weak correlation was also found between global DS3 and lyso‐Gb3 (*ρ* = 0.380, *P* = .025).

### 3.5 Hierarchical linear regression

Table 4 displays the results of hierarchical linear regression analyses where demographic variables, disease severity score and self‐reported symptoms were introduced into the model in a stepwise manner, while using SF‐36 component scores (PCS and MCS) as outcomes.

**TABLE 4:** Factors associated with physical and mental quality of life in patients with Fabry disease. Hierarchical linear regression with bootstrapping

	SF‐36 physical component summary (PCS)			SF‐36 mental component summary (MCS)		
	*β*	SE	*P*	*β*	SE	*P*
**Model 1**	**Adjusted *R* ^2^ = 0.332**			**Adjusted *R* ^2^ = 0.076**		
Age	−0.146	0.121	.235	0.105	0.096	.278
Sex	2.811	3.903	.455	4.654	2.851	.118
Education >12 y	16.464	4.101	**.001**	−4.072	2.816	.156
**Model 2**	**Adjusted *R* ^2^ = 0.488**			**Adjusted *R* ^2^ = 0.121**		
Age	0.027	0.108	.815	0.176	0.095	.084
Sex	−1.593	3.939	.702	2.834	3.251	.385
Education >12 y	11.710	4.538	**.019**	−6.037	3.434	.099
Global DS3	−0.950	0.330	**.009**	−0.393	0.301	.176
**Model 3**	**Adjusted *R* ^2^ = 0.661**			**Adjusted *R* ^2^ = 0.555**		
Age	−0.044	0.088	.600	0.137	0.076	.091
Sex	−0.910	3.364	.786	2.142	2.201	.345
Education >12 y	8.477	3.783	**.040**	−5.450	2.473	**.043**
Global DS3	−0.583	0.327	.079	−0.025	0.211	.895
Fatigue (VAS‐F)	−1.849	0.510	**.002**	0.179	0.443	.694
Depression (HADS‐D)	−0.437	0.415	.278	−1.752	0.308	**.001**

*Note*: With the two SF‐36 component scores (PCS and MCS) as outcomes, demographic variables (age, sex, education) were first included (model 1), then FD‐severity (Global DS3) (model 2) and finally depression (HADS‐D) and fatigue (VAS‐F) (model 3).

Abbreviations: HADS, Hospital Anxiety and Depression Score; VAS, visual analog scale.

Bold values indicate significant associations by the <0.05 threshold.

Background demographic factors explained 33% of variations in PCS, but almost none of the variation in MCS (model 1). Education >12 years was significantly positively associated with PCS (*β* = 16.464, *P* = .001).

When disease severity was included (model 2), 49% of the variation in PCS was explained. By each point increase in DS3, PCS was reduced by 1 point (*β* = −0.950, *P* = .009). DS3 score was not significantly associated with MCS (*β* = −0.393, *P* = .176).

In our fully adjusted model (model 3) 66% of the variation in PCS was explained. Education level (*β* = 8.477, *P* = .040) and VAS‐F (*β* = −1.847, *P* = 0.002) were the most influential factors, while depression was not associated with physical HRQOL. However, HADS‐D was significantly associated with mental HRQOL (*β* = −1.752, *P* = .001), rendering the fully adjusted model explanatory for 56% of the variation in MCS. Of note, higher education was associated with *lower* MCS (*β* = −5.450, *P* = .043).

### 3.6 Sex analyses

There was no significant difference in overall HRQOL between the sexes. The cohort was split by sex to investigate possible differences between the individual strength of the chosen explanatory variables (Suppl. Table 3). DS3 score might be a stronger predictor of physical HRQOL in males, while fatigue seems to weigh more for females. However, the small sample size prohibits firm conclusions.

## 4 DISCUSSION

In this study of individuals with FD, we investigated associations between HRQOL and demographic factors, disease severity, fatigue and depression. Higher education was strongly associated with better physical quality of life. Though disease severity (DS3) was associated with PCS, fatigue presented as the strongest indicator of physical HRQOL in multivariate analyses. Depression, assessed by the HADS score, was the only factor convincingly associated with mental HRQOL. Our models explained two thirds of the variation in physical HRQOL and approximately half of the variation in mental HRQOL. As might be expected in a multisystem disease like FD, several important determinants of HRQOL were interrelated.

### 4.1 HRQOL in a Norwegian Fabry cohort

The males participating in our study scored significantly lower in all SF‐36 domains than the general male population in Norway, while females scored lower than their female counterparts in the domains for general health and social functioning.

Pre‐ERT reports have shown that untreated males with FD have low HRQOL, their score profiles being in the range of patients with AIDS or severe hemophilia.^17^, ^20^ A more recent multicenter survey study including 311 FD patients of both sexes, of which two thirds received ERT, confirmed that Fabry patients had reduced HRQOL compared with the general population, with a mean PCS score of 41.7 and a mean MCS score of 48.7.^49^ Other studies have presented comparable results.^16^, ^50^ In today's era of ERT, Norwegian Fabry patients present with similar SF‐36 scores as patients with FD in other parts of the world.^19^, ^49^, ^51^, ^52^

### 4.2 Demographic factors and HRQOL

Age did not associate with physical (PCS) or mental (MCS) HRQOL in this study, contrary to some reports.^8^, ^16^ However, these data do not allow for evaluations of individual changes in HRQOL with increasing age. Since FD is X‐linked, implying more severe disease manifestations in males, we expected a sex difference in HRQOL. Low study power might be one reason why Role emotional domain was the only domain in which females scored significantly higher than males. However, in general population HRQOL studies, women tend to report *lower* life satisfaction than men,^53^, ^54^, ^55^ Norwegian females being no exception.^43^ Alternatively, the lack of convincing sex differences may reflect that genetic disease represents a shared burden in the whole family. For example, individuals knowing that they are genetically predisposed for future breast cancer are prone to experience deficits in HRQOL.^56^ Though physical symptoms may be scarce in many female heterozygotes, they are frequently mothers of affected children and caregivers for relatives with serious Fabry manifestations, which is likely to affect quality of life in several ways.^57^

As seen in other populations,^58^, ^59^, ^60^ including a Norwegian general population sample,^61^ higher education was significantly associated with better physical HRQOL. Education may be an advantage for acquiring knowledge and building motivation for lifestyle changes, which may alleviate symptoms and limit comorbidity. Of note, higher education was significantly associated with *lower* mental HRQOL, an association that seems previously undescribed. One might speculate that a deeper understanding of FD could increase worry or pessimism concerning potential disease progression in oneself or relatives.

### 4.3 Disease burden

The DS3 combines objective measures of Fabry‐related organ manifestations and subjective factors like gastrointestinal symptoms, pain and self‐perceived burden of disease. Not surprisingly, the global DS3 score could explain a significant share of the variation in physical quality of life in our study. Surrogate measures of disease severity, like enzyme activity and lyso‐Gb3 levels seemed also associated with PCS, though significance was not reached using *P*‐value <.001 (Table 3).

Notably, correlations between DS3 and mental quality of life (MCS) were absent. The patients' ability to adapt their expectations throughout their illness experience could be an important explanation, as well as the “disability paradox,” meaning that disabled individuals report good HRQOL because they focus on coping strategies and positive emotions.^62^

Among the organ‐specific DS3 domains, the cardiac domain seemed most strongly associated with physical HRQOL. This is in line with Gold et al. who in 2002 identified cardiac problems as a major determinant of HRQOL in FD.^20^ Cardiac disease in FD is largely due to hypertrophic cardiomyopathy, and symptoms on exertion often stem from diffuse microangiopathy due to sphingolipid deposits rather than coronary arteriosclerosis.^63^ As much as 75% of our patients reported some limitation in daily activities (NYHA class ≥2), and 25% reported dyspnea NYHA class 3 to 4. Parallel to the results from heart failure studies,^64^, ^65^ we found NYHA class to be strongly associated with physical HRQOL, underscoring the importance of optimizing the treatment of cardiac Fabry manifestations, including prophylaxis, stabilization and symptom alleviation. It should be noted, however, that dyspnea is nonspecific and does not always indicate Fabry cardiac disease; for example, shortness of breath at exertion may also result from pulmonary involvement of FD.^66^

The effect of ERT on life satisfaction in FD is uncertain,^16^, ^25^ and treatment status did not correlate with SF‐36 scores in our cohort. There is a possibility that Fabry‐specific therapy has only modest benefit in this domain. However, indication bias is frequently present, in that ERT is offered to those who are more seriously affected. This being the case also in our work, a potential treatment effect might not be picked up.

### 4.4 Pain

The DS3 PNS subdomain includes a crude pain assessment, but the use of more differentiated instruments to evaluate pain in lysosomal storage disease is encouraged.^33^, ^67^ In FD, most publications have focused on the BPI as a tool to evaluate ERT treatment effects.^24^, ^68^ We found BPI severity and interference scores to be age independent and correlate significantly with disease burden (DS3). This is in line with a recent report of robust correlations between BPI and MSSI scores in Brazilian patients with classic FD.^19^ Though multicollinearity prevented us from evaluating BPI together with DS3 in the final regression model, we observed strong associations between BPI pain interference and the physical domains of SF‐36 in both sexes. More modest correlations between BPI and HRQOL were reported in children with FD.^69^

### 4.5 Fatigue

Chronic fatigue is a complaint in about 50% of patients with FD.^52^, ^70^ Participants in the current study reported symptoms likely to be clinically significant with a mean VAS‐F of 6.9, notably higher than patients with rheumatoid arthritis whose mean score was 4.21.^71^ Excessive daytime sleepiness is independently associated with PCS in FD.^70^ In our study, increasing fatigue was significantly associated with reduced physical HRQOL, also in multivariate analyses. Unfortunately it seems to be one of the most difficult FD manifestations to manage, as there is little evidence of ERT reducing fatigue in treated patients.^49^

### 4.6 Anxiety and depression

Fabry patients of both sexes have reduced mental or emotional HRQOL.^25^, ^72^ An association between depression (HADS‐D) and mental HRQOL was clearly present in our study, explaining >50% of the variation in MCS. Our results are in harmony with a recent Brazilian report.^19^ Prevalence of depression in FD is reported in the range 15% to 62%, but is most likely underdiagnosed.^6^, ^35^, ^73^ Compared with a general Norwegian population sample,^74^ our Fabry patients of both sexes reported an increased burden of psychiatric symptoms. Anxiety scores above the threshold signaling clinical affective disease was especially frequent when compared with reference data,^75^ but none of our patients were receiving psychopharmacologic treatment.

Laney et al. showed that FD patients, particularly females, were affected by decreased social‐adaptive functioning, challenges which were significantly associated with anxiety and depression.^29^ As pain, fatigue, fear of the future and self‐perception of poor health are likely to be significant triggers of psychological distress in FD, we recognize the difficulty in determining if psychiatric disease in these patients is due to organic cerebrovascular disease or a more secondary phenomenon.^73^ We saw significant correlations between HADS depression score and both BPI interference and fatigue in our cohort. Whatever the etiology may be, given the magnitude and implications of psychiatric symptoms in FD, an increased focus on diagnosis and treatment of depression is warranted.^19^, ^76^

Fatigue, pain, depression and anxiety present as important determinants of HRQOL in several patients groups, for example, patients with stroke,^77^ systemic lupus erythematosus^78^, ^79^ and cancer,^80^ indicating that the interrelationship between these symptoms and their impact on HRQOL may be rather universal observations.

#### 4.6.1 Strengths and limitations

The clinical follow‐up design involving multidisciplinary medical expertise is a major strength of this study. However, the low sample size, a general challenge in orphan disease studies, restricts us from drawing firm conclusions. Some selection bias in patients willing to participate is unavoidable, such that more serious disease manifestations, psychological issues or fatigue might be more (or less) frequent among nonresponders.

The Fabry DS3 conveys a snapshot of the clinical status of the individual patient, preventing an evaluation of the clinical course over time. While the newly developed FAbry disease STability indeX (FASTEX)^81^ could have been useful for the evaluation of disease progression, data from multiple time points were available for only 67% of participants.

Also, BPI scores give a snapshot of pain experience. Several study participants reported that this questionnaire failed to give a true picture of their general pain burden related to FD. Recently translated into English,^82^ the Würzburg Fabry Pain Questionnaire^33^ might prove a useful tool, but a validated Norwegian translation is not yet available. Another limitation to be acknowledged is that the HADS questionnaire has not been validated for use in FD.

Though represented as one single item in the DS3 score, GI‐symptoms, a major challenge to the well‐being of many Fabry patients, were not investigated specifically in this study. Of note, our research group has work in progress, aiming to shed light on the association between abdominal symptoms and HRQOL in FD.

## 5 CONCLUSIONS

Individuals with FD in Norway have reduced HRQOL compared to the background population. The disease‐specific severity score DS3 is useful not only to evaluate and grade clinical disease manifestations but also as an indicator of physical HRQOL. DS3 is moderately correlated with pain related to daily functioning. However, it does not take into account chronic fatigue and psychological distress, factors which in this study were shown to be important determinants of physical and mental HRQOL, respectively. Future research on HRQOL in FD should include strategies on how to alleviate psychological and mental distress and cope with challenges of daily life.

## CONFLICT OF INTEREST

The results reported in the current manuscript should not lead to financial gain or loss for any organization or company from which the authors have received fees/salaries. Hege K. Pihlstrøm and Ketil R. Heimdal have received honoraria for work as consultants for Amicus Therapeutics and Sanofi Genzyme. Trond G. Jenssen has received honoraria for work as a consultant for Takeda and Sanofi Genzyme. Charlotte von der Lippe, Mina S. Weedon‐Fekjær, Birgitte L. Bjerkely, Kristin Ørstavik, Per Mathisen, Dag O. Dahle and Olga K. Solberg declare that they have no conflict of interest, apart from grants received by NKSD for conduction of the study.

## AUTHOR CONTRIBUTIONS

H.K. Pihlstrøm and S. Sigurdardottir: concept, design, data collection, database generation, data analysis and interpretation, writing and revising the manuscript. M.S. Weedon‐Fekjær: concept, design, data collection, interpretation of results, writing and revising the manuscript. B.L. Bjerkely: concept, design, interpretation of results, final design of all tables, writing and revising the manuscript. C. von der Lippe: concept, design, interpretation of results, writing/revising the manuscript. K. Ørstavik: design, database generation, interpretation of results, writing/revising the manuscript. P. Mathisen, T.G. Jenssen and O.K. Solberg: concept, design, data collection, interpretation of results, writing/revising the manuscript. K. Heimdal: concept, design, database generation, interpretation of results, writing/revising the manuscript. D.O. Dahle: study design, data collection, interpretation of results, writing and revising the manuscript.

## DETAILS OF ETHICS APPROVAL

All protocols and methods were approved by the Norwegian Regional Committee for Medical Research Ethics in South‐Eastern Norway, REK‐SørØst (permit no. 31513), and the Institutional Data Protection Officer.

## INFORMED CONSENT

All procedures followed were in accordance with the ethical standards of the responsible committee on human experimentation (institutional and national) and with the Helsinki Declaration of 1975, as revised in 2000. Informed consent was obtained from all patients for being included in the study. The consent forms are securely stored and will be made available on request.

## Supporting information


**APPENDIX S1**: List of abbreviations

**Supplementary Table 1** *GLA*‐mutations in 36 patients from 19 different families constituting the study population

**Supplementary Table 2** Pain location in patients with Fabry disease (n = 36)

**Supplementary Table 3** Hierarchical linear regression. Predictors of physical quality of life in men and women with Fabry disease.

## Data Availability

Norwegian ethical and legal restrictions prevent us from uploading data to public repositories or including the full dataset as Supplementary Material. Norwegian Fabry patients belong to a relatively small group, and very little personal data would be needed in order to indirectly identify individual study participants. We have been in dialogue with the Data Protection Authority of Oslo University Hospital in this matter. Access to a limited version of the dataset containing selected variables may be made available on request.
