# Supplementary material for: Health‐related quality of life in Norwegian adults with Fabry disease: Disease severity, pain, fatigue and psychological distress
Source: JIMD Rep. 2021 Jul 16;62(1):56–69. doi: 10.1002/jmd2.12240 (PMC8574186; doi:10.1002/jmd2.12240)
Supplement: Supplementary file 1 — APPENDIX S1: List of abbreviations [file JMD2-62-56-s004.docx]

APPENDIX: LIST OF ABBREVIATIONS

| BPI  ECG  ERT  FASTEX  FD  Gb3  HADS  HRQOL  IR  Lyso-Gbs  MCS  MRI  MSSI  NYHA  PCS  SD  SF-36  VAS  α-GAL | Brief Pain Inventory  electrocardiogram  Enzyme Replacement Therapy  FAbry disease STability indeX  Fabry Disease  Globotriaoscylceramide  Hospital Anxiety and Depression Scale  Health-related quality of life  Interquartile range  Globotriaosylsphingosine  Mental Component Summary  Magnetic Resonance Imaging  Mainz Severity Score Index  New York Heart Association  Physical Component Summary  Standard deviation  Short-form Health Survey  Visual analogue scale  α-galactosidase |
| --- | --- |
