# Supplementary material for: Health‐related quality of life in Norwegian adults with Fabry disease: Disease severity, pain, fatigue and psychological distress
Source: JIMD Rep. 2021 Jul 16;62(1):56–69. doi: 10.1002/jmd2.12240 (PMC8574186; doi:10.1002/jmd2.12240)
Supplement: Supplementary file 2 — Supplementary Table 1 GLA‐mutations in 36 patients from 19 different families constituting the study population [file JMD2-62-56-s001.docx]

**Supplementary table 1**

*GLA*-mutations in 36 patients from 19 different families constituting the study population

| PATIENT | GENE | ZYGOZITY | FAMILY | EXON | DNA | PROTEIN | PHENOTYPE** |
| --- | --- | --- | --- | --- | --- | --- | --- |
| 1 | GLA | Hemizyg | 1 | 7 | c.1024C>T | p.(Arg342*) | Classical |
| 2 | GLA | Heterozyg | 2 | 2 | c.334C>T | p.(Arg112Cys) | Female |
| 3 | GLA | Hemizyg | 2 | 2 | c.334C>T | p.(Arg112Cys) | Classical |
| 4 | GLA | Hemizyg | 2 | 2 | c.334C>T | p.(Arg112Cys) | Classical |
| 5 | GLA | Heterozyg | 3 | 7 | c.1024C>T | p.(Arg342*) | Female |
| 6 | GLA | Heterozyg | 4 | 5 | c.658C>T | p.(Arg220*) | Female |
| 7 | GLA | Hemizyg | 5 | 1 | c.124A>C | p.(Met42Leu) | Classical |
| 8 | GLA | Hemizyg | 6 | 7 | c.1082G>C | p.(Gly361Ala) | Classical |
| 9 | GLA | Heterozyg | 7 | 7 | c.1196G>A | p.(Trp399*) | Female |
| 10 | GLA | Heterozyg | 7 | 7 | c.1196G>A | p.(Trp399*) | Female |
| 11 | GLA | Hemizyg | 8 | 1 | c.124A>C | p.(Met42Leu) | Attenuated/indetermined |
| 12 | GLA | Hemizyg | 9 | 6 | c.901C>T | p.(Arg301*) | Classical |
| 13 | GLA | Heterozyg | 9 | 6 | c.901C>T | p.(Arg301*) | Female |
| 14 | GLA | Heterozyg | 9 | 6 | c.901C>T | p.(Arg301*) | Female |
| 15 | GLA | Heterozyg | 10 | 1 | c.124A>C | p.(Met42Leu) | Female |
| 16 | GLA | Hemizyg | 11 | 1 | c.124A>C | p.(Met42Leu) | Attenuated/indetermined |
| 17 | GLA | Hemizyg | 12 | 6 | c.902G>A | p.(Arg301Gln) | Attenuated/indetermined |
| 18 | GLA | Hemizyg | 13 | 7 | c.1082G>C | p.(Gly361Ala) | Attenuated/indetermined |
| 19 | GLA | Heterozyg | 13 | 7 | c.1082G>C | p.(Gly361Ala) | Female |
| 20 | GLA | Hemizyg | 14 | 4 | c.548G>T | p.(Gly183Val) | Classical |
| 21 | GLA | Heterozyg | 14 | 4 | c.548G>T | p.(Gly183Val) | Female |
| 22 | GLA | Heterozyg | 14 | 4 | c.548G>T | p.(Gly183Val) | Female |
| 23 | GLA | Heterozyg | 14 | 4 | c.548G>T | p.(Gly183Val) | Female |
| 24 | GLA | Heterozyg | 15 | 7 | c.1229C>A | p.(Thr410Lys) | Female |
| 25 | GLA | Heterozyg | 15 | 7 | c.1229C>A | p.(Thr410Lys) | Female |
| 26 | GLA | Heterozyg | 15 | 7 | c.1229C>A | p.(Thr410Lys) | Female |
| 27 | GLA | Heterozyg | 15 | 7 | c.1229C>A | p.(Thr410Lys) | Female |
| 28 | GLA | Hemizyg | 15 | 7 | c.1229C>A | p.(Thr410Lys) | Classical |
| 29 | GLA | Heterozyg | 16 | 1 | c.124A>C | p.(Met42Leu) | Attenuated/indetermined |
| 30 | GLA | Hemizyg | 17 | Intron 5 | c.802-2_802-3del | p.? | Classical |
| 31 | GLA | Heterozyg | 18 | 7 | c.1082G>C | p.(Gly361Ala) | Female |
| 32 | GLA | Hemizyg | 18 | 7 | c.1082G>C | p.(Gly361Ala) | Attenuated/indetermined |
| 33 | GLA | Hemizyg | 18 | 7 | c.1082G>C | p.(Gly361Ala) | Attenuated/indetermined |
| 34 | GLA | Heterozyg | 18 | 7 | c.1082G>C | p.(Gly361Ala) | Female |
| 35 | GLA | Heterozyg | 18 | 7 | c.1082G>C | p.(Gly361Ala) | Female |
| 36 | GLA | Heterozyg | 19 | 7 | c.1082G>C | p.(Gly361Ala) | Female |

**Classical phenotype: males with blood enzyme activity <2% of reference and Fabry-related clinical symptoms from childhood/adolescence
