# Supplementary material for: Health‐related quality of life in Norwegian adults with Fabry disease: Disease severity, pain, fatigue and psychological distress
Source: JIMD Rep. 2021 Jul 16;62(1):56–69. doi: 10.1002/jmd2.12240 (PMC8574186; doi:10.1002/jmd2.12240)
Supplement: Supplementary file 3 — Supplementary Table 2 Pain location in patients with Fabry disease (n = 36) [file JMD2-62-56-s002.docx]

| **Body region** | N | Frequency |
| --- | --- | --- |
| Head  Neck  Jaw  Shoulder region  Chest  Abdomen  Upper back  Lower back  Elbow region  Wrists/hand/fingers  Hip  Thighs  Knees  Calves  Ankles /feet | 7  4  1  8  5  9  7  6  8  15  7  3  11  9  18 | 19%  11%  3%  22%  14%  25%  19%  17%  22%  42%  19%  8%  31%  25%  50% |

Supplementary table 2. Pain location in patients with Fabry disease (n=36)

Self-reported pain in the last 24 hours.

Brief Pain Inventory (BPI) questionnaire
