# Supplementary material for: Health‐related quality of life in Norwegian adults with Fabry disease: Disease severity, pain, fatigue and psychological distress
Source: JIMD Rep. 2021 Jul 16;62(1):56–69. doi: 10.1002/jmd2.12240 (PMC8574186; doi:10.1002/jmd2.12240)
Supplement: Supplementary file 4 — Supplementary Table 3 Hierarchical linear regression. Predictors of physical quality of life in men and women with Fabry disease. [file JMD2-62-56-s003.docx]

| Suppl. table 3. Hierarchical linear regression. Predictors of physical quality of life in men and women with Fabry disease. | | | | | | | | | |
| --- | --- | --- | --- | --- | --- | --- | --- | --- | --- |
|  | **SF-36 Physical component summary (PCS)**  **Men** | | |  | | **SF-36 Physical Component Summary (PCS)**  **Women** | | |  |
|  | ***β*** | **SE** | ***p*** |  | | ***β*** | **SE** | ***p*** |  |
| Model 1 Adjusted R²= 0.279 | | | | **Adjusted R²=0.390** | | | | | |
| Age  Education >12 y | -0.380  15.690 | 0.240  5.921 | 0.073  **0.020** |  | | -0.005  19.568 | 0.170  6.343 | 0.988  **0.010** |  |
| Model 2 Adjusted R²= 0.708 | | | | **Adjusted R²=0.360** | | | | | |
| Age  Education >12y  Global DS3 | -0.001  8.086  -1.138 | 0.155  5.218  0.292 | 0.995  0.159  **0.004** |  | | 0.030  18.066  -0.296 | 0.177  11.238  1.224 | 0.852  0.117  0.669 |  |
| Model 3 Adjusted R²= 0.770 | | | | **Adjusted R²=0.641** | | | | | |
| Age  Education >12 y  Global DS3  VAS Fatigue | 0.048  5.640  -1.015  -1.291 | 0.130  4.257  0.312  0.636 | 0.628  0.230  **0.017**  0.066 | |  | -0.162  11.715  0.221  -2.739 | 0.154  7.686  0.801  0.897 | 0.255  0.115  0.611  **0.017** |  |

Abbreviations: VAS, visual analogue scale
